# Transition services in 22q11 deletion syndrome: Hit or miss

**DOI:** 10.1016/j.hctj.2025.100120

**Published:** 2025-09-18

**Authors:** Sophie Ayoub, Sandra Meier, Cheryl Cytrynbaum, Ann Swillen, Luzius A. Steiner, Holly Carbyn, Bernice S. Elger, Eva De Clercq

**Affiliations:** a Institute for Biomedical Ethics, University of Basel, Basel, Switzerland; b Department of Psychiatry, Dalhousie University, Halifax, NS, Canada; c IWK Health Centre, Halifax, NS, Canada; d Division of Clinical & Metabolic Genetics and the Department of Genetic Counselling, the Hospital for Sick Children, Toronto, Canada; e Department of Molecular Genetics, University of Toronto, Toronto, Canada; f Center for Human Genetics, University hospital UZ Leuven, Leuven, Belgium; g Department of Human Genetics, KU Leuven, Leuven, Belgium; h Department of Anesthesia, University Hospital Basel, Basel, Switzerland; i Department of Clinical Research, University of Basel, Basel, Switzerland; j Center for legal medicine, Faculty of Medicine, University of Geneva, Geneva, Switzerland

**Keywords:** 22q11 deletion syndrome, Rare disease, Transition, Transitional care pathway, Ethical principles, Decision-making

## Abstract

**Introduction:**

22q11 deletion syndrome (22q11DS) is a rare genetic condition and the most common microdeletion in humans. The symptomatology is broad and variable between individuals. It is usually diagnosed in childhood and requires a multidisciplinary lifelong approach including a structured transitional care process. This study therefore seeks to enrich our understanding of transitional care for individuals with 22q11DS by exploring the insights from healthcare professionals (HCPs) from Europe and Canada with multidisciplinary backgrounds.

**Methods:**

This interview study was part of a larger mixed-methods research project, with the qualitative component concentrating on gathering insights from HCPs involved in patient care through semi-structured interviews. We conducted qualitative thematic analysis while focusing on transitional care within families caring for a child with 22q11DS from the perspective of HCPs.

**Results:**

The 20 HCPs interviewed came from diverse professional backgrounds and all had clinical experience with 22q11DS. Our analysis of the data identified three primary themes. Our participants emphasized the inadequacy of the transitional care system, despite its critical importance. HCPs tried their best to overcome the challenges by relying on longer follow-up with their patients, with some even merging children and adult services. There was a shared desire among HCPs for a structured transitional care plan, better access to adult services for 22q11DS, and a more integrated, collaborative approach.

**Conclusion:**

This study highlights the inadequacy of transitional care for individuals with 22q11DS and their families, showing a need to better align policies and clinical practice. Filling the gap between existing policies and the desired transitional care services is essential. The latter includes: an appropriate longitudinal transition plan, strong psychological support not only for the individual with 22q11DS but also for their family, a coordination system, and accessible adult services with expertise on 22q11DS.

## Highlights

• Aligning policies and clinical practice is necessary for transitional care in 22q11 deletion syndrome.
• Ethical considerations are important including respect of autonomy and supported autonomy.
• A longitudinal transition plan with strong psychological support is critical.
• A lack of adult services with expertise on 22q11DS has to be addressed.

## 1 Introduction

22q11 deletion syndrome (**22q11DS**) is the most common microdeletion syndrome with a prevalence of 1:2148 in diagnosed individuals.^1^ Its symptomatology is wide-ranging and can affect all physiological systems. The most common features include congenital heart defects, palatal anomalies, immune and endocrine malfunctions and a wide range of neurodevelopmental delays and psychiatric disorders with a high risk of developing psychosis later in life.^2^ The impact of 22q11DS varies among individuals, with symptoms ranging from mild to severe.^3^

Most patients with 22q11DS typically require lifelong, coordinated multidisciplinary care from various healthcare professionals (**HCP**s).^4^ As a result, a structured transitional care process to ensure continuity between pediatric and adult healthcare services is warranted.^5^, ^6^ Transitional care is a purposeful, planned process that involves the deliberate coordination and support of adolescents and young adults as they move from child-focused to adult-focused healthcare systems. It should be supported by a structured and proactive approach, not a sudden handoff, with an emphasis on three major components: planning, transfer, and integration.^7^, ^8^, ^9^ Without proper transition planning, patients and their families may face gaps in medical management, loss of specialized care, and increased health risks during critical life stages. A well-executed transition process guarantees seamless, well-rounded, and coordinated care that aligns with the changing needs of patients while addressing the expectations of families and healthcare providers.^6^

A recent report by Irish clinicians working with individuals with 22q11DS emphasized how a well-structured transition strategy can facilitate better symptomatic management, improve quality of life, and ensure continuity of care. At the same time, it contributes to the efficient utilization of healthcare resources, and reduces long-term burdens on medical services.^10^ Gold et al. ^11^ provide important conceptual insights on healthcare transitions for adolescents and adults with 22q11DS based on their own experiences. The authors emphasize the importance of a successful transition to adult healthcare in ensuring optimal long-term well-being and quality of life for individuals with 22q11DS and their families.

Despite the well-documented heightened risk of various medical and psychiatric conditions persisting from childhood into adulthood for young individuals with 22q11DS, transitional care is still lacking.^12^ Based on lived experiences of individuals with 22q11DS, a lack of proper communication between child and adult health services ^13^ and a gap in a long-term coordinated approach, multidisciplinary collaboration, and personalized care was present.^12^ Research with caregivers has also shown dissatisfaction with services when transitioning from pediatric to adult care.^14^

Until now, most studies on transitional care in 22q11DS has concentrated on perspectives of patients and families. Although HCPs play a central role in the success of transition programs, currently studies from the perspective of HCPs are limited to theoretical reflections on existing obstacles and potential solutions, rather than being grounded in empirical data.^11^

This study, therefore, aims to understand the challenges of transitional care for individuals with 22q11DS by exploring the insights from HCPs from Europe and Canada with multidisciplinary backgrounds and analyze them through an ethical lens.

## 2 Methods

The data for this interview study were collected as part of a broader mixed methods research project that aimed to improve the psychosocial well-being of children aged 3–15 years with 22q11DS and their families. The qualitative part of this study aimed to capture the caregiving experiences of HCPs by conducting semi-structured interviews following an interview guide (Supplementary Material).

### 2.1 Participants and sampling

The sample for this part of our interview study consisted of HCPs from Europe and Canada, with at least one year of work experience in the field of 22q11DS (e.g. Genetics, Cardiology, Immunology, Endocrinology, Speech and Language Pathology, Otolaryngology, Psychiatry, Plastic Surgery and Orthopedics), caring for children (3–15 years) with 22q11DS and their families. Additional inclusion criteria include: 1. Can read, write, and understand English, French or German, 2. Can provide informed consent and 3. Be available for a 40–60-minute interview (online or in person).

We aimed for sufficient richness and depth to meaningfully address our research questions, reached around interview HCP18 (with 2 extra interviews). No pre-study interaction occurred between the interviewer and the participants All participants were recruited through purposeful sampling: project collaborators (N = 2) followed by snowballing (N = 18). Snowballing consisted of two phases: some HCPs (N = 10) were referred by other participants and then, to reach the target sample size, 21 professionals, who were board members of 22q11DS key organizations (22q11 Europe, 22q society) and who had valid contact details, were contacted via email. The email summarized the research project and invited them to participate in an interview. Of those who responded, 6 met the eligibility criteria and were interviewed (N = 6), and they subsequently provided contact details for 2 additional interviewees (N = 2). A written informed consent was obtained from all HCPs.

### 2.2 Data collection

Interviews were conducted starting October 19, 2022, through April 21, 2023, via the digital platform Zoom by the same researcher (SA), a female medical doctor trained to conduct qualitative research. Interviews were held in English. The purpose of the study, benefits and minimal risks were explained, after which respect for privacy and confidentiality were discussed. The interviewer followed an interview guide that focused on HCPs’ caregiving experience, perceived needs of families, available support services including transitional care, perceived impact of the condition on families and their coping experience, and employed targeted probing questions adapted to each encounter.

### 2.3 Data analysis

All interviews were recorded with consent, transcribed verbatim, and inductively coded using the qualitative analysis software MAXQDA 24 to facilitate structured data analysis. To ensure anonymity and confidentiality, any personal or situation-specific details that could lead to the identification of the interviewee were de-identified following transcription. Coding began concurrently with transcription. Thematic analysis (TA) by Braun and Clark 15, 16 was used by the above-mentioned researchers to analyze the empirical data. Thematic analysis is a qualitative research method used to identify, analyze, and interpret themes within data.^15^ Following Braun and Clarke’s six-phase approach to reflexive thematic analysis, SA and EDC first familiarized themselves with the data by transcribing and repeatedly reading all the interviews. They then generated initial codes collaboratively from two interviews to develop a shared understanding of the data and analytic approach. SA subsequently coded the remaining interviews alone, with ongoing reflexive discussions with EDC to refine the codes and ensure depth of interpretation. SA then constructed potential themes, which were reviewed, defined and finalized in collaboration with EDC. Both researchers produced the first draft and integrated co-authors contributions’ and comments in the final report.^17^

### 2.4 Credibility and reflexivity

To enhance credibility, findings were reviewed by two independent researchers. To enhance reflexivity, SA remained aware of how her professional experiences (as a clinician not in rare diseases (**RD**s)) and her own assumptions could influence data analysis. To minimize bias, she actively reflected on her ideas and remained engaged in this ongoing process throughout the collection and analysis of the data. Through ongoing reflexive engagement, including reading, discussion and team discussions, the researcher remained aware of how her own perspectives, experiences and assumptions may influence the analytical process. Rather than seeking to bracket subjectivity, the analysis aimed to interpret participants’ accounts meaningfully while being transparent about the researcher’s role in developing themes.^18^

### 2.5 Ethics approval

The study was approved by the Ethics Committee of the University of Basel for research with human subjects. The study was conducted in compliance with the protocol, the current version of the Declaration of Helsinki, the ICH-GCP, and ISO EN 14155 (as far as applicable) as well as all national legal and regulatory requirements. The data were stored in accordance with the General Data Protection Regulation (GDPR) on a secure university server and were only accessible to the research team.

## 3 Results

In this paper, we report on results coming from data collected through 20 interviews with HCPs (16 females, 4 males). The interviews lasted between 40 and 77 min in length. Participating HCPs came from diverse disciplines: genetic counseling, psychology, psychiatry, ENT (ear, nose, and throat) surgery, pediatrics, speech-language pathology, clinical immunology, nursing, and occupational therapy. Most of them had extensive experience working with children with 22q11DS and their families: 55 % had more than 20 years of experience, 25 % had between 10 and 20 years of experience, and 20 % had less than 10 years of experience. 15 participants worked in a clinic specifically for patients with 22q11DS and the rest worked either in an university hospital or a general clinic, all located either in Canada or Europe.

**Fig. 1:**
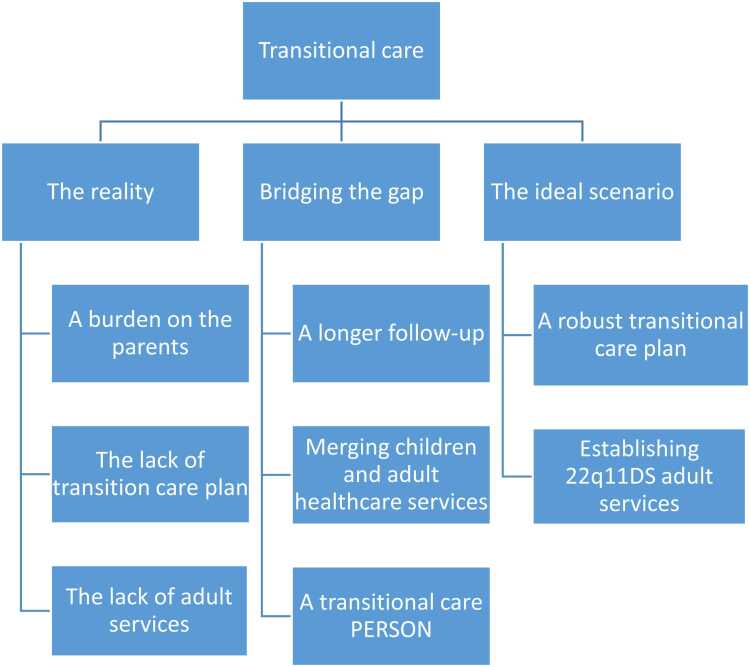
Themes and subthemes.

Analysis of the results identified three primary themes, centered around transitional care in different contexts: 1. The reality of existent (or non-existent) transitional care 2. How HCPs are bridging the reality to the ideal transitional care and 3. The ideal transitional care settings (see Fig. 1).

We used illustrative quotes in tables (see Table 2, Table 3, Table 4) to expand on each topic.

**Table 1:** Characteristics of participants.

Years of experience		Specialty	Work institution
1–10	HCP4	Psychologist	University hospital
	HCP9	Child psychiatrist	General clinic
	HCP17	Occupational therapist	General clinic
	HCP19	Pediatrician	22q clinic
10–20	HCP8	Genetic counselor	22q clinic
	HCP10	ENT^(1)^ surgeon	University hospital
	HCP14	Psychologist	22q clinic
	HCP16	SLP^(2)^	22q clinic
	HCP18	Psychologist	22q clinic
20–30	HCP1	Genetic Counselor	22q clinic
	HCP3	Psychologist	22q clinic/university hospital
	HCP5	ENT surgeon	University hospital
	HCP6	SLP	22q clinic
	HCP7	Nurse coordinator	22q clinic
	HCP12	Pediatrician	22 g clinic
	HCP13	Clinical immunologist	General clinic
	HCP15	Psychiatrist	22q research clinic
	HCP20	SLP	22q clinic
30 +	HCP2	SLP	22q clinic
	HCP11	Pediatrician	22q clinic

(1) Ear,nose and throat- (2) Speech-Language Pathologist

**Table 2:** Quotes for the theme 1: The reality of existent (or non-existent) transitional care.

**A burden on the parents**
*“I’ve been working in transitions since like 10 years and there are always these policy directors that are like ah the transition plan, the transition plan and I don't think it is fair that it should be the parents because are the parents supposed to know all of the resources of the adult system, from where? How are they supposed to know all of that?” (HCP17, occupational therapist)*
*“I mean I think the transition it's hard to be an adult for many of these individuals, it's hard to manage with the adult life, I've not heard it so much with parents of young children but more when they are almost adults or are young adults and so on, I think it comes up more to discussion.” (HCP20, speech-language pathologist)*

**The lack of transitional care plan**
*“(…) I think one of the difficulties in this condition is that the child may be under a range of specialties all of which have to be transitioned, but none of it is done in any systematic way. So, I would say once the child becomes 18, what care looks like probably is yearly appointments with whoever is still following them up. So maybe a yearly appointment with the cardiologist or with the neurologist or whatever, but there is no united picture (…) Transition services in other specialties are hit and miss: some have really good transition clinics like diabetes clinics do, the cleft team does, but some you hit 16 and you end up in the adult services, and that's basically it.” (HCP14, psychologist)*
*“We've had some very positive feedback about that (a transition care plan) as opposed to saying ok today is your last appointment, next time you're going to go and to see this new team. So at least having that one day, when we bring all families together to inform of how the transition will go, has been essential. And we've had some very positive feedback about that. I think there's a lot more we can do to enhance the process, but time constraints and resources are always a challenge in healthcare so we continue to work towards enhancing that process.” (HCP1, genetic counselor)*
*“Typically around the time they turn 18, right now after COVID we are not doing it anymore, but we used to actually have a proper transition day, where we bring a group of children with their parents into the clinic and we would do an assessment of the patient in the morning and then an education piece where we take the parents and the kids separately: the parents would learn about navigating resources in the adult world and the kids would get asked questions about what is 22q and learn a little about how to advocate for their own health needs and then the adult clinic would actually do a presentation to them and meet them, and bring them to the adult clinic, and we walk down there and get a tour of the clinic. So that was a nice way of doing it because there's less fear and worry and they become familiar with the adult world.” (HCP8, genetic counselor)*

**The lack (and bad performance) of adult services for 22q11DS**
*“The problem is that they don't really have anyone to transition them to I think that's the tricky thing.” (HCP14, psychologist)*
*“I think this transition is really difficult and it doesn't work at all. I mean child care and child's hospital they are really good to take care of the families and these special illnesses which the adult hospital or adult care do not do.” (HCP20, speech-language pathologist)*
*“(…) When they transition to adult services, there is no coordination, it all disappears. And also what is challenging is that when they are 18 and we have to refer them from the pediatric services and we have nobody, no experts in the adult health care system who can take over, I would have to refer them to a GP (general practitioner).” (HCP13, clinical immunologist)*
*“There are 3 or 4 transitions during early adulthood where you're going from your pediatric providers to your adolescence care providers, to young adult providers, to your final adult providers. And I don't know how it is elsewhere, but resources disappear with each step, like you don't hear of multidisciplinary comprehensive assessment teams that would spend a full day in the adult world of the neurodevelopmental population, it's much more scarce there” (HCP9, child psychiatrist)*
*“At the 22q clinic, they help families with the transition to the adult services; they have transition as part of their service and there's an adult 22q clinic just across the street. So, I think there is good support for families and adolescents coming to the adult system because the 22q staff works with them to make referrals, to help them with the paper work, to introduce them to the right people (…) It's a challenge maybe for families who do not live in (city) to find someone to help them access these services, so it's not as easy perhaps for them to come to (city) to see.” (HCP4, psychologist)*

### 3.1 Theme 1: the reality of existent (or non-existent) transitional care (Table 2)

All HCPs agreed that transitioning to adulthood is a complex process for families of children with 22q11DS, especially with an ideal transitional care being non-existent. The unavailability and the non-performing 22q11DS adult services made the situation even more complicated.

#### 3.1.1 A burden on the parents

All participants acknowledged that the transitional phase is challenging for individuals with 22q11DS and their families to deal with the adult life, especially when not well supported. Additionally, the lack of a transition care plan placed a burden on the parents, forcing them to take responsibility for guaranteeing access to appropriate adult services, without enough knowledge to do so. *(HCP17, occupational therapist-HCP20, speech-language pathologist)*

#### 3.1.2 The lack of transitional care plan

The complexity of this transition phase arises from the condition requiring multiple specialties, each of which necessitates its own transition process. While some departments or specialties have established a transition process to support the families throughout this period, others have not. Stated differently, the quality of the transition depends on which expertise you are seeking. But a unified and systematic approach under a transition plan is missing. *(HCP14, psychologist)*

Part of a transition care plan, defining one day to inform parents about the transition process has been helpful. However, even HCPs, who have that process in place for their families, complained about time constraints, lack of resources, or the COVID pandemic impeding a smooth transition. *(HCP1, genetic counselor-HCP8, genetic counselor)*

#### 3.1.3 The lack (and bad performance) of adult services for 22q11DS

The complexity is not solely due to the absence of a structured transitional care plan by itself. Rather, transition is a process that inherently requires the involvement of many parties. If one party is absent, the entire process becomes unsustainable. For example, many HCPs emphasized that, contrary to the pediatric healthcare setting, adult services specialized in 22q11DS are largely missing. *(HCP14, psychologist-HCP20, speech-language pathologist)*

When available (very rarely), adult services lacked expertise in managing the care of these families, and for this reason, participants worried about the transition phase as well. They described how families are well taken care of in the pediatric healthcare system. Then, when they reach a certain age, they have to transfer to adult services. There, professionals with expertise on 22q11DS are rare, and coordination seems nonexistent, making referrals to general practitioners, who might be unable to take care of individuals with complex needs, their only option. In other words, specialized adult 22q clinics are practically non-existent. Therefore, adult services are typically provided by independent clinicians with a lack of coordinated care. *(HCP13, clinical immunologist)*

The poor performance of adult services can be due to misallocation of resources (focused usually on the early years) that seem to disappear with each chronological step in the care of children, more so for the population with neurodevelopmental challenges. *(HCP9, child psychiatrist)*

Moreover, even when adult services were available, they were not accessible to everyone and, as is often the case, they were limited in rural areas compared to urban. Families coming from rural areas struggled to find appropriate and easily accessible adult services for 22q11DS, especially when transitioning from pediatric services. Finding a specialist in adult services who could support them locally was challenging; as a result, these families had to travel to receive the necessary care. *(HCP4, psychologist)*

### 3.2 Theme 2: how HCPs are bridging the reality to the ideal transitional care (Table 3)

All participants agreed on the importance of a transitional care but also on its absence. Their responses to this reality varied depending on the availability of human, financial and time resources to fill in the gap in transitional care.

**Table 3:** Quotes for the theme 2: How HCPs are bridging the reality to the ideal transitional care.

**A longer follow-up of patients**
*“The clinic really went up to early adult life so late teen that is unusual for the hospital because in (country), there is transfer of children and their families to adult services between 14 and 16.” (HCP11, pediatrician)*
*“(…) So we could extend through our craniofacial cleft palate program to 22–23 years of age because individuals who have cleft palate need some orthognathic surgery jaw surgery and they don't do that until an individual has reached skull maturity so in boys then it might be 21–23, females it's a little bit earlier so we can follow them if needed but the only reason I would only follow them is because they had a surgery and we just want to make sure that in fact the surgery is successful.” (HCP2, speech-language pathologist)*
*“We are quite fortunate to work in a private foundation so we don't have like a very clear limit so we don't need to stop at like 18, which is good because as it is the case for a lot of other conditions, transitioning at 18 does not make any sense. Usually we try to see the families until they are like in the more stabilized period until at least 25–30.” (HCP18, psychologist)*
*“I hold on to them (in my private practice) until they turn 19, because no one kicked them out. But appreciatively, (…) the other institutions are big psychiatric institutions and have made transition age program so they'd follow children in these programs until 25 which is great (…) because that's a real vulnerable window between 16 and 22.” (HCP9, child psychiatrist)*
*There's interest in building capacities, for developmental programs in the city and I know one group working on it looking at what kind of transitional supports to include because really the whole pediatric system cuts off at 18 and it's a big problem especially if a lot of the (psychological) problems have onset at age 18 for 22q11DS.” (HCP9, child psychiatrist)*

**Merging children and adult services for 22q11DS**
*“I think at our center, genetics and the 22q11 consultation center there, they still see them even when they are adults. But normally, feeding and speech and language are not so much an issue anymore so I don't need to see them (in adulthood) but they'd still be followed up by the genetic and 22q team.” (HCP16, speech-language pathologist)*
*“It's a very variable syndrome so it really depends there are patients who do not need me actually and there are others that I follow up for years until they are 12 or even older. It's not a fixed program it depends on their needs (…) If they don't need follow up anymore for their speech, I don't see them unless there is still some issue going on (…) And (name) from genetics follows them longer and she refers to me if there is an ENT problem.” (HCP10, ear-nose-throat surgeon)*
*“Well, for the medical part, they transition to the adult services but the clinical genetics team stays the same. It doesn't matter how old you are for clinical genetics. Which means my youngest patient with 22q11DS is 10 months old and the oldest one is 74, who is a grandfather (…). One has to see the full picture of a patient across different stages in life, otherwise, one is completely biased, (…) it starts at the beginning when you are referred to a clinical genetic center where you get care as a whole (…). And I even think the longer I follow up with this population, the more I see the diversity also (…). They are definitely vulnerable on certain points, but on the other hand they also have these amazing things like very good in music, in nature… (HCP3, psychologist)*

**A transitional care person**
*“We don't really have an adult 22q clinic at the moment, we have an amazing immunologist who works in the hospital who was fighting tooth and nail to somehow work out what to do with these young people as they go forward and she actually is single handedly keeping lots of these people closely on her books to monitor them but she sees them for immunology issues but then her letters involve numerous other things because she knows the condition so well, so she might write letters to many other people in adult services saying you have to monitor this and this and this but really there is no 22q clinic in adult services.” (HCP14, psychologist)*
*“(As adults), they have to find an outpatient clinic where they live, and I should refer them there and have a discussion with them (HCPs of the outpatient clinic). Then I write a referral letter and a longer summary of their condition and how it is today and even information about what to do and what to follow, what information they can get and I give them information about the new guidelines published. And I even often print it out to send it together with a referral letter and a summary with the patient.” (HCP12, pediatrician)*
*“If there is a need for transition, then we try to make a transition to colleagues but there is not really like an adult clinic for 22q in (name of city). So usually if this is necessary, we refer to like people in private practice who know a little bit about the syndrome, and then we still remain available for any advice or information, that's usually how we do it.” (HCP18, psychologist)*
*“Short answer is no (I am not involved in the transition). But you know if, for example, if there's somebody who's 19 or 20 who still requires surgical intervention, then I'm involved in transitioning them to one of the adult surgeons that I know does that type of surgery.” (HCP5, ear-nose-throat surgeon)*
*“To talk about transition, I will give a very good example. So there is a patient who is currently under my care, he must be about 18 and in addition to 22q, he is very complex (…) He was managed by (name of clinic) in (city), but when he reached 18, he just appeared as a referral; there was no real handover. Fortunately, because of my background, I helped them negotiate the system (…), the only thing not well covered was his mental health support. Now if he hadn't come to me, if he had gone to an average clinic or physician, I do not know that they would have had the knowledge to support him.” (HCP14, psychologist)*

#### 3.2.1 A longer follow-up of patients

However, the age at which children transitioned out of pediatric services varied across centers. In some cases, the need for a longer follow-up is not fixed, but rather assessed with regard to the underlying medical condition or to the sex. This is due to the difference in the patient’s medical needs and the physiology between girls and boys. *(HCP11, pediatrician-HCP2, speech-language pathologist)*

In other cases, interviewees tried delaying the transition to a later stage because the stress on the individuals is lower (unlike during adolescence and early adulthood). Some HCPs considered it a privilege to be working for a private institution and be able to delay transition for children with 22q11DS and other conditions for which setting the age of 18 unconditionally for transition within healthcare systems seemed inappropriate. *(HCP18, psychologist-HCP9, child psychiatrist)*

Adding a developmental (community) program to reinforce capacities of teenagers and young adults to a transitional program allowed them a smoother transition to adulthood rather than a sudden cut-off in support services. This empowers individuals with 22q11DS to better face the psychological challenges that might arise during this period of time. *(HCP9, child psychiatrist)*

#### 3.2.2 Merging children and adult services for 22q11DS

A few participants decided to merge pediatric and adult services into one big department led by the genetic team. The latter is considered the main consultant, coordinating the care longitudinally, and then medical referral happens separately in each specialty if the affected individuals (when they become adults) are still in need of care in that specialty. *(HCP16, speech-language pathologist-HCP10, ear-nose-throat surgeon)*

The rationale for merging adult and child services is to adopt a holistic approach to care, ensuring better follow-up and continuity of treatment. The interviewees emphasized that this integration shifted their perspective on patient care, as failing to observe patients across multiple life stages can lead to biases and hinder a comprehensive understanding of their needs. The participants noted that the “diversity” in patient presentations becomes more apparent when patients are followed longitudinally, allowing healthcare providers to offer more informed and effective care. (*HCP3, psychologist)*

#### 3.2.3 A transitional care person

Other solutions for imperfect conditions of transition (not having appropriate adult services) focused on defining two parties communicating and collaborating well: the pediatric and adult services. Often, a person driven by a sense of duty, had to replace an absent transition team. For example, in one clinic, one HCP made recommendations to professionals in the adult services on what to monitor and how to follow-up with their patients since there is no adult clinic for 22q11DS. *(HCP14, psychologist)*

Similarly, in other institutions, the responsibility falls on a single HCP who takes on the additional task of preparing patients for transition. This includes writing a long summary and referral letter addressed to their new formal caregiver, or referring them to colleagues they know, easing on the transition process and making sure the communication during this phase is adequate and always possible in case of a need for advice or clarification. *(HCP12, pediatrician-HCP18, psychologist-HCP5, ear-nose-throat surgeon)*

As mentioned before, the lack of awareness and expertise in the adult healthcare world affects the quality of the transition and the long-term follow-up, especially in cases where the professionals working with adults with 22q11DS are unaware of medical challenges related to the condition. In this example of care experience, a HCP had to step in to fill in the gap in the handover of a complex case from the pediatric services. However, participants noted that this was not the perfect scenario since the psychological support was still missing. *(HCP14, psychologist)*

### 3.3 Theme 3: the ideal transitional care settings (Table 4)

Almost all HCPs wished a better structure for transitional care for families of children with 22q11DS. This included an appropriate transitional care plan and available adult services for 22q11DS.

**Table 4:** Quotes for the theme 3: The ideal transitional care settings.

**A robust transitional care plan**
*“I think all of that (a transitional care plan) in place would be really good, so families aren't looking for something that they had previously, that's not there in the adult population. So it's a good thing if they have that but I'm not sure that they have that everywhere. But transitioning the adulthood where these patients are now accountable for their own care, it is also important to have a team to help through that system.” (HCP7, nurse coordinator)*
*“(…) it's probably changed since COVID but they (the 22q team) really support these young adults and you know they're setting them up before they transition so they know what's going to happen (…), they often book all their appointments and go over with them, just to support them, to say this is your new team, you know they're going to look after you, those are the people that you talk about your concerns and worries with, how you can access care and how you can go forward with your life.” (HCP7, nurse coordinator)*
*“I find if we do it too early the kids the teens don't get as much out of it, but anyway there's also other planning that's helpful for adults early, because in terms of disability supports that are accessible through different federal programs, and parents need to learn about those earlier so that they can apply to get some additional financial support for the children.” (HCP1, genetic counselor)*
*“What we try to do is have parents start understand that transition happens very early like the transitioning has to happen in stages, so we do some education with parents about giving their children an opportunity to develop those independence skills, even if it takes longer and even if it's frustrating and easier to put their clothes for them or to brush their teeth for them. We help parents see that as an opportunity to get their kids to practice independence even from a very young age, going to the store and learn how to pay for something themselves, take a bus to school like even if they have to do it with them for ten times but eventually they can do it by themselves. We're working on that right now, to have something a bit more formal like developing a checklist for ages and stages and what parents can do, what kids can practice and what we can do to help kids and parents do that.” (HCP8, genetic counselor)*
*“We took the initiative and partnered with the adult clinic and what we realized quite quickly is that transition is not a single event right? it's a process and you need to prepare individuals for transition and I think one of the things that we could do better that we're trying to do, is to develop sort of check ins periodically, to talk about how you can help parents to support their child move to being increasingly independent and responsible for their care. So, for young adults with 22qDS they continue to need a fair bit of support for most individuals, but there's a way that you can give them more independence. And a lot of the families are afraid or not sure how they do that (…) So I think we need to do a better job at starting younger with those type of tips and guidance.” (HCP1, genetic counselor)*
*“(…) Then of course it is all the problems with being independent and I don't know how this should be organized but to find a place to live and work and I think those who have a more severe problem again, who have an intellectual disorder let's say, they have get more help with this specific types of working places or places to be on the day and also get help with (…)” (HCP20, speech-language pathologist)*

**Establishing adult services for 22q11DS**
*“The great thing about the transition clinic is the smooth transition to the next phase but if you don't have a good setup in the next phase it doesn't really work. So what you need is almost an equivalent 22q clinic in adult services that would be the idea of it you just pass everything over.” (HCP14, psychologist)*
*“Certainly for the older patients as they're transitioning out of ((name of clinic) that (transition care plan) is something that has been expressed, I'm not sure if you're familiar that we have the adult's clinic that we can transition our families to, I think that has made a huge difference in terms of families’ comfort level when time come to leave (name of clinic) and transition out, knowing that same of kind team approach to care and access to really expert knowledge in 22q is going to continue.” (HCP6, speech-language pathologist)*
*“I think they need, should need a specific physician taking care of them who could be their doctor, and who can help them to go to different places and also I think so many of them need some kind of psychiatric help, which they don't get enough and also people who know about these specific problems.” (HCP20, speech-language pathologist)*
*“The biggest challenge is that we don't really know what happens to these individuals once they're adults. Actually we just recently did a study, (person) did a study looking at obstructive sleep apnea in individuals with adults with 22q and one of the things that we've found is if they've had a pharyngeal flap which a surgery to correct nasal sounding speech in childhood it increases their risk to have obstructive adult onset obstructive sleep apnea (…) And we now know we have to counsel any individual with 22q who've had a pharyngeal flap about obstructive sleep apnea (…) that's really important about something that goes on in adulthood, and we should know about as pediatric team so that we can make sure that we're being proactive.” (HCP2, speech-language pathologist)*

#### 3.3.1 A robust transitional care plan

Ideally, HCPs wish to see a real transition care plan installed for families of children with 22q11DS and better adult services. A transition care plan, firstly and ideally should be in place, regardless of the availability of specialized adult services and more so if the latter is absent. Adult patients become accountable for their own care, thus need advice on how to handle the new challenges. *(HCP7, nurse coordinator)*

Secondly, a close-to-perfect support during the transition phase, as per our participants, has to include the opportunity for families of children with 22q11DS to meet their future team-carer and build confidence in the new team who will take over their care and help them to access support services in the adult world. *(HCP7, nurse coordinator)*

Additionally, HCPs insisted on the importance of a good timing of the transition, not being too early so the individuals with 22q11DS can benefit more if they are more mature and prepared, nor too late so that they (individuals with 22q11DS) do not miss opportunities for access to care. *(HCP1, genetic counselor)*

Moreover, a few HCPs noted that the ideal transition should not happen in one step. It should always be seen as a progressive process that allows enough time to develop the necessary skills to move forward to the adult world. Transition should begin very early in life, with small daily tasks to coach children with 22q11DS on how to become independent. The perfect scenario according to this participant, would be to set official checklists for families to follow. Our participants also mentioned the importance of fostering independence in the young adults with 22q11DS for a smoother transition, especially those who have difficulty organizing their practical needs and those who have intellectual challenges in their daily life. *(HCP8, genetic counselor-HCP1, genetic counselor- HCP20, speech-language pathologist)*

#### 3.3.2 Establishing adult services for 22q11DS

Finally, the majority of our participants recognized the urgency of establishing adult services clinics specifically for individuals with 22q11DS and their families. Offering equivalent support to that of the children’s clinics in term of knowledge of the professionals involved, the holistic care approach and the variety of services offered, including psychological support deemed currently insufficient but necessary to this vulnerable population. *(HCP14, psychologist-HCP6, speech-language pathologist-HCP20, speech-language pathologist)*

However, a scenario where pediatric and adult 22q11DS clinics exist does not necessarily mean we’ve achieved perfection if there is insufficient collaboration between the two clinics. As stated by our interviewees, longitudinal follow up of pediatric patients with respect to outcomes is critical to appropriately inform an evidence-based approach. *(HCP2, speech-language pathologist)*

## 4 Discussion

### 4.1 Discussion of the results

The present study provides a valuable contribution to the literature on transitional care and 22q11DS by exploring the perspectives of HCPs. Since the transitional period is characterized by a high level of stress,^13^ and HCPs are primary supporters of families navigating 22q11DS, their insights are essential for improving care. Our most important finding was that despite a lot of publications written on transitional care in general, on RDs 19, 20, 21 and specifically on 22q11DS,^10^, ^11^ the actual transitional care system lacks many key components. Developing a healthcare transition program involves getting in touch with stakeholders and refining the reality to pre-existing theories.^22^ Got Transition's Six Core Elements,^23^ a widely used, structured approach to health care transition, is endorsed by major medical organizations and tailored to different practice types. The six elements include: developing policies and guides, tracking and monitoring of the progress of the patient, assessing their readiness for transition, planning of and the actual transfer of care and finally the completion of transition. We noticed that clinicians genuinely aimed to incorporate these core elements into the process, recognizing their potential to improve patient care and outcomes. They seemed to focus on the readiness for transition (i.e. by working on the independence of individuals with 22q11DS) and the structure and completion of the transition. However, despite their intentions, the integration often fell short due to systemic barriers, time constraints, and limited resources. This is similar to the findings of a scoping review on core components of successful transitions from child to adult mental health services in which these core elements seemed to be tied to the clinicians in comparison to the first core element (on policy making) tied to the administrative teams.^24^ This still raises the question of how to support the experts into integrating existing frameworks on transitional care (i.e. Got Transition) into their daily routine care.^25^

Our findings highlight the key challenges in transitional care, HCPs’ perspectives on the ideal system they envision, and the strategies they employ to compensate for its absence. Embedded within these themes are important ethical considerations that positively impact the transition from pediatric to adult services.^26^ Additionally, the lack of transitional care is an ethical breach by itself.^27^ These ethical considerations include the redefinition of autonomy, the principle of justice, and the imperative to avoid harm.

First, (individual) autonomy is defined as self-rule that is free from both controlling interference by others and limitations that prevent meaningful choice.^28^ In pediatrics, for minors, this is far more complicated because parents (in most cases) have the legally binding decision-making power and because balancing the family’s, parent’s and siblings’ wellbeing adds another layer of complexity. But minors’ participation (vs. individual autonomy) in decision-making is encouraged in clinical practice and research.^29^, ^30^, ^31^ For example, a recent report from the World Health Organization (WHO) provides new guides on assessing and supporting adolescents' capacity for autonomous decision-making.^32^ The report emphasized shared decision-making and people-centered care that respects the perspectives of young people, their families, and communities. Recognizing that minors may hold values different from adults, the guidance addresses the ethical tension between protecting their well-being and supporting their autonomy and encourages careful deliberation when ethical principles conflict.

This nuanced application of autonomy in pediatrics applies to transitional care services and based on the results of this study for children with 22q11DS in particular, it is evident that supported autonomy, facilitated by caregivers (parents) and professionals (including HCPs), is crucial. The transition process is often challenging due to anxiety about the future and the emotional difficulty of leaving familiar pediatric healthcare providers. To ease this process, our participants emphasized the importance of accompanying not only affected individuals but also families through the transition process. Educating both patients and their families is a key responsibility for all HCPs involved.^11^ Educating the children themselves and preparing them for the next step has been widely discussed from a medical (i.e. taking over their healthcare planning ^33^), educational (acquiring the necessary skills to manage their financial life ^10^) and psychosocial aspect (i.e. navigating independent living challenges ^10^). Coaching programs are thus clearly needed to guide young people with 22q11DS to transition more easily into adulthood.^34^ Our results on educating and providing support for the families, particularly caregivers, align with existing literature on 22q11DS which highlights expert recommendations on family inclusion in transitional care and the necessity of caregiver support as perceived by affected individuals.^12^ Informal caregivers/families play a crucial role in the transition from pediatric to adult care, yet many find it difficult to step back and encourage their child’s independence in healthcare decisions.^13^

Second, the principle of justice is particularly relevant when addressing the challenges faced by children with a complex and RD such as 22q11DS. These children have an urgent need for continuity of care during and after transition. Our research participants emphasized that a well-structured transitional care plan, an unfragmented approach, and increased awareness among adult healthcare providers regarding the needs of individuals with 22q11DS and their families were essential components of equitable healthcare.

Respecting justice means access to care for everyone regardless of their age or location. Our participants reported a fragmented care approach for 22q11DS in the adult services and even a total absence. This is not surprising since the literature discusses the difficulty of finding experts on many RDs (i.e. 22q11DS) in pediatric and adult services,^13^, ^35^ and criticizes the fragmented care approach.^36^ Physicians who specialize in adult care typically lack confidence and are less inclined to manage complex conditions that begin in childhood or to work with patients diagnosed with intellectual disabilities.^37^

We also noted through our interviews that transition and the quality of care in adulthood depended strongly not only on which specialty patients are seeking but also on where. Our participants mentioned that children with complex needs and those living in rural areas seem to have more difficulty accessing transitional care. In one study in the US, families with lower incomes, often living in rural areas where access to specialized care was limited, were more likely to move in search of better healthcare options.^38^ The key aspects of such a continuity of care according to our results include: (1) transitional care team/coordinator, (2) collaboration between pediatric and adult care providers, (3) conceiving transition as a process rather than a one-time event.

A transition care plan was mentioned in many of our interviews and was related to a transitional care team that can support the families along this phase. This is similar to what we saw in the literature as a coordinator person or team could be the link between specialties and between pediatric and adult services to avoid fragmentation of care and ensure a better collaboration.^39^

Ideally, our participants hope for collaborative meetings between pediatric and adult care providers. Literature shows this enables adolescents and their families to adapt to the adult care system while facilitating knowledge exchange among professionals.^11^ Additionally, there remains considerable debate regarding the appropriate timing of transition. Our findings suggest that setting a fixed chronological age at which children transfer from pediatric to adult services does not satisfy most HCPs regardless of their work location or area of specialty. Instead, there was a consensus on the need for a progressive transition process rather than a single, fixed event.

Given that developing condition-specific programs for each potential clinical presentation of 22q11DS (including neurodevelopmental disorder) may be impractical, a more feasible approach may involve core transitional care supported by tailored modules.^5^, ^27^

Finally, the avoidance of harm cannot be achieved without appropriate psychological support, which was highlighted as a key concern by our participants. Chronic conditions, particularly RDs, can be isolating for families who experience psychological distress 40, 41 and cause depression and anxiety to caregivers and the adolescents affected by them.^42^, ^43^ Ensuring adequate emotional and mental health support during the transition period is critical to mitigate potential negative impacts on both patients and their families ^44^(reference accepted not yet published), especially since this period of life has been associated with the worsening of symptoms for any disease (prevalent and rare diseases).^5^, ^45^ Additionally, with 22q11DS is associated with a higher risk of developing psychosis and other psychiatric illnesses. Thus, it is crucial to be familiar with signs and symptoms of mental health disorders to provide adequate psychological support especially when approaching adolescence.^46^, ^47^

### 4.2 Practical implications

**Fig. 2:**
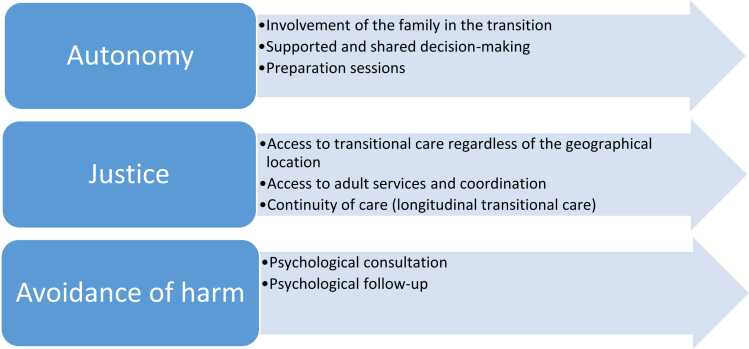
Practical implications through an ethical lens.

This study offers several practical implications (see Fig. 2), highlighting how the findings can be applied in real-world contexts. The results underscore actionable insights that may inform policy, practice, or further research.

### 4.3 Limitations

Not all participants had extensive expertise in transitional care but, we still believe our results capture the main aspects of care. We were unable to include some specialties (i.e. social care) nor experts in adult services, however, we included the most commonly involved disciplines, where HCPs have a broad understanding of the care needs in the period of transition to adolescence/adulthood. These insights are essential for understanding the specific transition care needs and challenges faced by families of children with 22q11DS.

## 5 Conclusion

This study puts the spotlight on the inadequacy of transitional care for individuals with 22q11DS and their families, highlighting the need to better align policies and clinical practice. Filling the gap between existing policies and the desired transitional care services is essential to strengthen the ethical foundation of these services. The latter includes: an appropriate longitudinal transition plan, strong psychological support, a coordination system, and available adult services with expertise on 22q11DS.

## Ethics approval and consent to participate

The project was approved by the Ethics Committee of the University of (blinded for peer review). The study was conducted in compliance with the protocol, the current version of the Declaration of Helsinki, the ICH-GCP, or ISO EN 14155 (as far as applicable) as well as all national legal and regulatory requirements. The data were stored in accordance with the General Data Protection Regulation (GDPR) on a secure university server and were only accessible to the research team. HCPs were sent an information sheet and a written informed consent was obtained.

## Funding

This study is part of an ERA-NET project. The work was supported by the Swiss National Science Foundation (eCARE 22q11 SNSF:10ER1C_203747), the Canadian Institute for Health Research (CIHR) Canadian Research Chairs (CRC) stipend [award number 950–232098] and Team Grant: Transnational Research Projects on Rare Diseases [grant number ERT 179613]. The funders are not involved in the collection, analysis or writing. The interpretation and opinions are those of the authors.

## CRediT authorship contribution statement

**Sophie Ayoub:** Writing – review & editing, Writing – original draft, Validation, Methodology, Investigation, Formal analysis, Data curation. **Eva De Clercq:** Writing – review & editing, Validation, Supervision, Methodology, Formal analysis, Conceptualization. **Bernice S. Elger:** Writing – review & editing, Visualization, Validation, Supervision, Resources, Project administration, Methodology, Funding acquisition, Conceptualization. **Holly Carbyn:** Writing – review & editing. **Luzius A. Steiner:** Writing – review & editing. **Ann Swillen:** Writing – review & editing, Resources. **Cheryl Cytrynbaum:** Writing – review & editing, Resources. **Sandra Meier:** Writing – review & editing, Resources, Project administration, Funding acquisition, Conceptualization.

## Consent for publication

Consent obtained with the institutional written consent.

## Declaration of Competing interest

The authors declare that they have no known competing financial interests or personal relationships that could have appeared to influence the work reported in this paper.

## Data Availability

All data generated or analyzed during this study are included in this published article.

## Footnotes

Appendix A Supplementary data associated with this article can be found in the online version at doi:10.1016/j.hctj.2025.100120.
